# Extracellular Enzyme Activities and Carbon/Nitrogen Utilization in Mycorrhizal Fungi Isolated From Epiphytic and Terrestrial Orchids

**DOI:** 10.3389/fmicb.2021.787820

**Published:** 2021-12-21

**Authors:** Zeyu Zhao, Shicheng Shao, Na Liu, Qiang Liu, Hans Jacquemyn, Xiaoke Xing

**Affiliations:** ^1^Key Laboratory of Bioactive Substances and Resources Utilization of Chinese Herbal Medicine, Ministry of Education, Institute of Medicinal Plant Development, Chinese Academy of Medical Sciences and Peking Union Medical College, Beijing, China; ^2^Xishuangbanna Tropical Botanical Garden, Chinese Academy of Sciences, Xishuangbanna, China; ^3^Department of Ecological and Environmental Engineering, Yunnan Forestry Technological College, Kunming, China; ^4^Department of Biology, Plant Conservation and Population Biology, KU Leuven, Leuven, Belgium

**Keywords:** orchid mycorrhizal fungi, extracellular enzyme activity, nutrient preference, orchid life forms, ecological adaption

## Abstract

Fungi employ extracellular enzymes to initiate the degradation of organic macromolecules into smaller units and to acquire the nutrients for their growth. As such, these enzymes represent important functional components in terrestrial ecosystems. While it is well-known that the regulation and efficiency of extracellular enzymes to degrade organic macromolecules and nutrient-acquisition patterns strongly differ between major fungal groups, less is known about variation in enzymatic activity and carbon/nitrogen preference in mycorrhizal fungi. In this research, we investigated variation in extracellular enzyme activities and carbon/nitrogen preferences in orchid mycorrhizal fungi (OMF). Previous research has shown that the mycorrhizal fungi associating with terrestrial orchids often differ from those associating with epiphytic orchids, but whether extracellular enzyme activities and carbon/nitrogen preference differ between growth forms remains largely unknown. To fill this gap, we compared the activities of five extracellular enzymes [cellulase, xylanase, lignin peroxidase, laccase, and superoxide dismutase (SOD)] between fungi isolated from epiphytic and terrestrial orchids. In total, 24 fungal strains belonging to Tulasnellaceae were investigated. Cellulase and xylanase activities were significantly higher in fungi isolated from terrestrial orchids (0.050 ± 0.006 U/ml and 0.531 ± 0.071 U/ml, respectively) than those from epiphytic orchids (0.043 ± 0.003 U/ml and 0.295 ± 0.067 U/ml, respectively), while SOD activity was significantly higher in OMF from epiphytic orchids (5.663 ± 0.164 U/ml) than those from terrestrial orchids (3.780 ± 0.180 U/ml). Carboxymethyl cellulose was more efficiently used by fungi from terrestrial orchids, while starch and arginine were more suitable for fungi from epiphytic orchids. Overall, the results of this study show that extracellular enzyme activities and to a lesser extent carbon/nitrogen preferences differ between fungi isolated from terrestrial and epiphytic orchids and may indicate functional differentiation and ecological adaptation of OMF to local growth conditions.

## Introduction

With >26,000 species, the Orchidaceae is one of the largest and most diverse plant families in the world. Orchids can be found in almost all terrestrial ecosystems except the Antarctic and extremely hostile environments such as deserts and tundra vegetation (Cribb et al., 2003; Roberts and Dixon, 2008; Swarts and Dixon, 2009). Compared with seeds of other plant families, orchid seeds consist of merely undifferentiated embryos and are too tiny to contain adequate nutrient reserves. As a result, most orchid seeds cannot germinate without fungi (Kauth et al., 2008) and hence nearly all orchids rely on mycorrhizal fungi to obtain nutrients to support seed germination and early seedling growth. Typical orchid mycorrhizal fungi (OMF) associating with green orchids involve members of the Tulasnellaceae, Ceratobasidiaceae, and Serendipitaceae (Smith and Read, 2008; Dearnaley et al., 2012; Jacquemyn et al., 2017). Because of their dependence on mycorrhizal fungi, OMF have been regarded as one of the important factors determining whether an orchid can occur in one habitat or not (McCormick and Jacquemyn, 2014), and they have also been linked to niche partitioning and coexistence of multiple orchid species (Jacquemyn et al., 2015; Xing et al., 2020).

Orchids have different life forms and occupy a wide range of niches. Orchid species can be divided into terrestrial and epiphytic/lithophytic species (Cribb et al., 2003). Over 70% of orchid species have an epiphytic life style and grow in tree canopies to obtain light (Gravendeel et al., 2004), but at the same time they have less access to nutrients and water (Chase, 1987). Terrestrial orchids, on the other hand, have their roots in the soil and therefore rely more on the prevailing water and nutrient conditions encountered in the soil. Because of these differences in environmental conditions, it can be expected that not only the mycorrhizal fungi associating with terrestrial orchids differ from those associating with epiphytic species (Martos et al., 2012; Xing et al., 2019), but that also their ecological functions differ (Leake and Cameron, 2012).

Orchid mycorrhizal fungi are saprophytic and secrete extracellular enzymes to break down complex macromolecules such as cellulose, hemicellulose, and lignin into smaller units that can be taken up by the cell for growth and assimilation. Extracellular enzymes are diverse and can be divided into, among others, hydrolases, lyases, oxidoreductases, and transferases (Linhardt et al., 1987; Volc et al., 1997; Emri et al., 2008; Bachhawat and Kaur, 2017; Falade et al., 2017). Because these compounds contribute to diverse ecological functions including resource acquisition, protection, competition, interspecific and intraspecific interactions, they represent important functional components in terrestrial ecosystems (Bennur et al., 2015; Banerjee et al., 2018; Zucconi et al., 2020).

The regulation and efficiency of extracellular enzymes differ strongly between fungal groups (Kohler et al., 2015). Wood decaying fungi evidently show high decaying capabilities, while mycorrhizal fungi, i.e., fungi that form a mutualistic symbiosis with plants, generally have lower decay abilities than their ancestral wood decayers (Kohler et al., 2015). Nonetheless, most mycorrhizal fungi have retained a unique array of plant cell wall degrading enzymes, which provide the fungi access to mineral nutrients either sequestered with dead plant cells or complexed with cell wall components (Read and Perez-Moreno, 2003; Kohler et al., 2015). Extracellular enzymes are produced by ectomycorrhizal (ECM) fungi and ericoid mycorrhizal fungi and their contribution to the symbiosis have been well-investigated (Colpaert and Van Laere, 1996; Burke and Cairney, 2002; Adeoyo, 2021; Lang et al., 2021). However, the extracellular enzymes in OMF have been less intensively examined and their ecological functions remain largely unknown.

Additionally, it has long been known that different fungi have different nutrient-acquisition patterns. In recent years, the exchange of nutrients between orchids and fungi has been well-investigated (Cameron et al., 2008; Stockel et al., 2014), while only few studies focused on nutrient acquisition patterns in OMF. Nurfadilah et al. (2013) assessed the accessibility of specific nutrients by comparing growth of OMF *in vitro* and found that each of the OMF accessed and effectively utilized a wide variety of nutrient compounds. Since OMF communities strongly differ between terrestrial and epiphytic orchids (Martos et al., 2012; Xing et al., 2019), it can be expected that nutrient-acquisition patterns of OMF and preference for certain carbon and nitrogen sources also differ between OMF from terrestrial and epiphytic orchids, but so far empirical evidence is scant.

In this study, we hypothesized that the mycobionts of epiphytic and terrestrial orchids show differences in extracellular enzyme activities and carbon/nitrogen preference because they grow in completely different environments. Specifically, because fungi isolated from epiphytes endure more light exposure and drought stress than fungi isolated from terrestrial orchids, antioxidant and drought resistance related extracellular enzymes should show higher activities in OMF of epiphytes than those of terrestrial orchids. While soils are rich in organic substances from leaf and wood litter, extracellular enzymes related to nutrient absorption and cellulose degradation should show higher activities in OMF of terrestrial orchids than those of epiphytes. To test these hypotheses, we isolated and identified 24 strains of Tulasnellaceae from 18 orchid species (8 terrestrial and 10 epiphytic species) that grow in the same area. Activities of five extracellular enzymes [cellulase, xylanase, lignin peroxidase, laccase, and superoxide dismutase (SOD)] were compared between fungi from terrestrial and epiphytic orchids. These enzymes were selected due to their close relationship with symbiosis and nutrient acquisition. Additionally, growth rates of these Tulasnellaceae strains on media containing 20 different carbon and nitrogen sources were also determined.

## Materials and Methods

### Sampling

Fungi were collected from both epiphytic and terrestrial orchids in the Menglun sub-reserve (21°41′N, 101°25′E), one of the sub-reserves of the national nature reserve established in Xishuangbanna, Yunnan province, China. This region is characterized by a semi-humid, tropical monsoon climate with annual temperatures varying between 15.1 and 21.7°C and annual rainfall between 1,196 and 2,492 mm. Xishuangbanna national natural reserve is well-known for its high diversity of orchid species, and at present more than 400 different orchid species have been identified in this area (Liu et al., 2015). In the Menglun sub-reserve, eight terrestrial and 10 epiphytic orchid species were sampled (Table 1). For each orchid species, root samples from at least five individual plants were collected. In total, 90 individual root samples were collected. When sampling, we collected 3–5 root segments (2 cm) from each individual without dislodging the plant to minimize damage. The root samples were stored in an icebox and transported to the lab. In the lab, roots that were slightly yellow or opaque and that had *Rhizoctonia*-forming fungal mycelia on the surface were selected and surface cleaned several times with sterile water to minimize contamination with soil fungi. The roots were then checked microscopically for mycorrhizal colonization and used for isolation of OMF.

**Table 1:** *Tulasnellaceae* spp. isolated from orchids with different life forms.

Fungal isolate	Sequence-based identification	Closest matches in GenBank (accession numbers)	BLAST match sequence		Host orchids	Life form
			Coverage (%)	Max ident (%)		
HN5	Tulasnellaceae sp.	Uncultured Tulasnellaceae clone 52-2 (KX587478)	100	94.33	*Arundina graminifolia*	Terrestrial
HN10	*Epulorhiza* sp.	*Epulorhiza* sp. Pca-QS-0-1 (GU166424)	98	98.48	*Calanthe triplicata*	Terrestrial
HN15	*Tulasnella* sp.	Uncultured *Tulasnella* clone PA163 (FJ786651)	97	90.74	*Calanthe triplicata*	Terrestrial
YC05	Tulasnellaceae sp.	Uncultured Tulasnellaceae clone 21-24 (KX587475)	74	81.64	*Liparis nervosa*	Terrestrial
YC08	Tulasnellaceae sp.	Uncultured Tulasnellaceae clone YN51-18 (KP053823)	100	98.98	*Liparis nervosa*	Terrestrial
70-1	Tulasnellaceae sp.	Uncultured Tulasnellaceae clone PD570 (GQ241842)	96	98.37	*Phaius flavus*	Terrestrial
70-2	Tulasnellaceae sp.	Uncultured Tulasnellaceae clone FM228.1 (JF691347)	79	88.21	*Phaius flavus*	Terrestrial
LI2	*Tulasnella* sp.	Uncultured fungus clone PHA4_3 (KC588915)	99	96.48	*Liparis nervosa*	Terrestrial
AS50	*Tulasnella* sp.	*Tulasnella* sp. isolate R-84 (MT611051)	96	98.30	*Acanthephippium sylhetense*	Terrestrial
LV1	Tulasnellaceae sp.	Uncultured Tulasnellaceae clone Di_Aga_3D3 (JX024734)	100	99.58	*Liparis barbata*	Terrestrial
PY4	Tulasnellaceae sp.	Uncultured Tulasnellaceae clone OTUA5_PlateKaro_1_F7 (JX649082)	100	97.81	*Geodorum densiflorum*	Terrestrial
LG32	*Tulasnella* sp.	*Tulasnella calospora* isolate Bpn179 (MG008683)	99	99.65	*Liparis odorata*	Terrestrial
BQ	Tulasnellaceae sp.	Uncultured Tulasnellaceae clone DFi-XL19 (JX545221)	100	99.42	*Liparis elliptica*	Epiphytic
BZ	Tulasnellaceae sp.	Uncultured Tulasnellaceae clone Di_Aga_3D3 (JX024734)	100	99.15	*Vanda brunnea*	Epiphytic
KY	*Tulasnella* sp.	*Tulasnella* sp. 140 (AY373281)	98	98.25	*Epigeneium amplum*	Epiphytic
YX	Tulasnellaceae sp.	Uncultured Tulasnellaceae clone C5C_2A (KC243942)	98	92.47	*Chiloschista yunnanensis*	Epiphytic
HN19	*Tulasnella* sp.	*Tulasnella deliquescens* (LC175331)	93	98.61	*Dendrobium nobile*	Epiphytic
GA1	Tulasnellaceae sp.	Uncultured Tulasnellaceae clone Di_Karo_4G2 (JX024731)	99	96.94	*Gastrochilus bellinu*	Epiphytic
CF12	Tulasnellaceae sp.	Uncultured Tulasnellaceae clone OTU43 (MH005882)	93	99.54	*Cleisostoma fuerstenbergianum*	Epiphytic
AR13	Tulasnellaceae sp.	Uncultured Tulasnellaceae clone FM105.1 (JF691200)	96	90.73	*Aerides rosea*	Epiphytic
OJ-3	Tulasnellaceae sp.	Uncultured Tulasnellaceae clone PCG4_ITS4_TUL (KM211335)	95	99.21	*Dendrobium chrysotoxum*	Epiphytic
OJ-4	Tulasnellaceae sp.	Uncultured Tulasnellaceae clone PCG5_ITS4_TUL (KM211336)	92	99.38	*Dendrobium chrysotoxum*	Epiphytic
DO-3	Tulasnellaceae sp.	Tulasnellaceae sp. strain SSCDO-4 (MH348613)	100	99.67	*Dendrobium cucullatum*	Epiphytic
OD-7	Tulasnellaceae sp.	Uncultured Tulasnellaceae clone 43-11 (KX587484)	100	97.67	*Dendrobium cucullatum*	Epiphytic

### Isolation and Identification of OMF

A scalpel and forceps were used to carefully scrape off root hairs and velamen in sterile water within Petri dishes. Then the root segments were sterilized by soaking them in 75% ethanol for 1 min, a 1% sodium hypochlorite solution for 1 min, and 75% ethanol for 30 s. Afterward, the roots were rinsed with sterile water three times. The sterilized roots were immersed in 5 ml sterile distilled water with 100 mg/ml streptomycin sulfate and 100 mg/ml potassium penicillin G. The pelotons were released by scraping the root segments gently with a dissecting needle and further cultured in the dark at 20°C for 16 h. After incubation of pelotons for 16 h, the pelotons with new growing hyphae were picked out and transferred to a potato dextrose agar (PDA) plate, followed by culturing in the dark at 20°C. After 5–7 days, a small number of hyphae was visible and then transferred to a new PDA plate.

Genomic DNA of the obtained fungal isolates was extracted using a modified DN14-CTAB Plant Genomic DNA Rapid Extraction Kit (Aidlab, Beijing, China) according to the manufacturer’s instructions. The internal transcribed spacer (ITS) region of the fungal nuclear ribosomal RNA gene was amplified using the universal primer pair ITS1/ITS4 (White et al., 1990). The ITS1 primer covers ITS1-5.8S-ITS2 from the 5′ and ITS4 covers the same area from the 3′. PCR was performed in 50 μl reaction mixtures and run as follows: initial denaturation at 95°C for 5 min, followed by 35 cycles of 94°C for 45 s, 50°C for 45 s and 72°C for 1 min, and a final elongation step at 72°C for 10 min. PCR products were checked by electrophoresis on 1% agarose gels. Target rDNA nucleotide sequence data were obtained by DNA sequencing (Taihe Biotechnology Co., Beijing, China) employing the same primers as for the PCR procedures. The obtained sequences were compared with sequences available in GenBank using the Basic Local Alignment Search Tool (BLAST; Altschul et al., 1990). After typical orchid mycorrhizal fungal taxa (Dearnaley et al., 2012) were identified, one strain from each taxon was randomly selected and used for extracellular enzyme activity assays.

### Determination of Extracellular Enzyme Activities

#### Fungal Cultures

The media used for fungal culturing were prepared differently for the distinctive performance of extracellular enzyme activities in different substrates, especially for enzymes that are induced by substrates. The preparation of cellulase and xylanase activity determination medium was based on Grujić et al. (2019) with some minor modifications. The specific formula per liter was as follows: 2.0 g NH_4_NO_3_, 1.0 g K_2_HPO_4_, 1.0 g NaH_2_PO_4_, 1.4 g (NH_4_)_2_SO_4_, 0.3 g Urea, 0.5 g MgSO_4_·7H_2_O, 2.0 g yeast extract, and 30.0 g wheat straw. Wheat straw was crushed by a grinder and then dried for 2 h at 105°C through an 18-mesh sieve. The prepared medium was divided into 250 ml conical vials at the ratio of 100 ml/vial and sterilized by autoclaving at 120°C for 25 min.

The medium used to assess lignin peroxidase and laccase activities was based on Fillat et al. (2016), with some modifications. The specific composition per liter was as follows: 10.0 g sucrose, 2.0 g asparagine, 2.0 g KH_2_PO_4_, 0.5 g MgSO_4_·7H_2_O, 1.0 g thiamine hydrochloride, 57 μl veratryl alcohol, 0.05 g CuSO_4_, 0.1 g CaCl_2_, 2.0 g wheat straw, 1.5 g nitriloacetic acid, 1.0 g NaCl, 0.5 g MnSO_4_·H_2_O, 0.1 g FeSO_4_·7H_2_O, 0.1 g CoCl_2_, 0.1 g ZnSO_4_·7H_2_O, 0.01 g AlKSO_4_·12H_2_O, 0.01 g H_3_BO_3_, and 0.01 g NaMoO_4_·2H_2_O. The wheat straw treatment method was similar as above. Because asparagine, thiamine, and veratryl alcohol cannot sustain under autoclaving, the mixture of these three components was sterilized *via* filtration through a 0.22 μm microporous membrane. Then the mixture was added to the rest of the components that had cooled down to room temperature after autoclaving at 120°C for 25 min and divided into 100 ml per 250 ml conical vial.

The medium that was used to assess SOD activity was based on information in Angelova et al. (1996) and adapted with some minor modifications: 3.0 g casein, 4.0 g soybean meal, 20.0 g glucose, 0.0029 g ZnSO_4_·7H_2_O, 0.0043 g FeSO_4_·7H_2_O, 0.5 g MgSO_4_·7H_2_O, 0.0013 g MnSO_4_·H_2_O, and 0.0011 g CuSO_4_. After autoclaving at 120°C for 25 min, the media were divided into 100 ml per 250 ml conical vial as described above.

The inoculation of 24 *Tulasnellaceae* spp. was carried out as follows: circular fungal inoculums (1 cm diameter) were cut from the outer circumference of an active fungal colony in the form of agar plugs. Afterward, the agar plugs were grounded into small fragments by a glass rod, which made the agar plugs more evenly distributed and accelerated the growth rate of inoculum in the new medium. Three inoculums were used in every conical vial, and for each fungus and each culture medium, four replicas were used. All 288 bottles of cultivation media were cultured in the dark at 20°C with collection of the fermentation liquid regularly (every 3 days), yielding for each bottle eight samples in total (at day 3, 6, 9, 12, 15, 18, 21, and 24). Sampling was carried out in a sterile environment, and 1 ml culture solution without mycelium was sucked up with a pipette as crude enzyme solution. The rest of the culture solution and fungal mycelium were sealed and continued to culture. The enzyme stock solutions were stored at 4°C until testing. Enzyme activities were measured spectrophotometrically (Infinite M200 PRO NanoQuant, Tecan, Swiss).

#### Determination of Cellulase

The filter paper was used as the substrate to measure the integral cellulase activity, which contains the activities of endoglucanases (endo-1,4-β-glucanases, EGs), cellobiohydrolases (exo-1,4-β-glucanases, CBHs), and β-glucosidases. The experimental methods largely follow the procedures described in Silveira et al. (2012) with some minor modifications. First, a glucose standard curve was drawn by boiling different concentrations of glucose standard solutions (1.0, 2.0, 3.0, 4.0, 5.0, 6.0, 7.0, 8.0, 9.0, and 10.0 mM) and 3,5-dinitrosalicylic acid (DNS) reagent (1%, w/v) for 5 min and subsequently measuring the absorbance at 540 nm wavelength after the solution had cooled down at room temperature. The concentration of glucose was then correlated to *A_540_* and the equation of the regression line was calculated. Next, 30 mg Waterman No. 1 filter paper (diameter 0.6 cm filter paper × 7 pieces), 600 μl 0.1 M sodium acetate buffer (pH = 5.0), and 0.5 ml crude enzyme solution were added into a 1.5 ml centrifuge tube and mixed evenly. The reaction system was placed in a constant temperature water bath at 50°C for 30 min, then boiled for 5 min to terminate the reaction, and finally cooled to room temperature to form the saccharification liquid. Lastly, the content of glucose in the saccharification solution was determined using the standard curve determined above. One unit of cellulase activity was defined as the amount of cellulase required to catalyze the production of 1 μmol reducing sugar per milliliter of reaction solution for 1 min.

#### Determination of Xylanase

Xylanase activity was measured by degrading beech xylan into reducing sugars, which were then detected by DNS reactions similar to cellulase determination. The experimental methods followed procedures outlined in Zhang et al. (2020) with some minor modifications. First, the content of reducing sugar of crude enzyme solution was detected before enzymatic hydrolysis. After that, 50 μl crude enzyme solution was mixed with 100 μl beech xylan solution (1%, w/v) followed by immersing the solution in a water bath at 40°C for 15 min. Then the solution was heated quickly in boiling water for 5 min to inactivate enzymes. After cooling to room temperature, the reducing sugar content of the mixture was detected by DNS reaction. Different reducing sugar content in the mixture solution and the crude enzyme solution was produced by xylanase hydrolysis reaction. One unit of xylanase activity was defined as the amount of xylanase required to catalyze the production of 1 μmol reducing sugar per milliliter of reaction solution for 1 min.

#### Determination of Lignin Peroxidase

We measured lignin peroxidase activity using methods outlined by Magalhães et al. (1996). More specifically, 144 μl crude enzyme solution, 8 μl 50 mM veratryl alcohol solution and 40 μl 0.5 M potassium tartrate solution were added to a UV-96-well plate (with UV transparent flat bottom). After that, 8 μl 10 mM hydrogen peroxide was added as a catalyst to start the reaction, and the absorbance change per minute was measured at 310 nm within the initial 3 min. One unit of lignin peroxidase activity was defined as the amount of lignin peroxidases required to produce 1 mol of veratraldehyde per milliliter reaction solution in 1 min.

#### Determination of Laccase

Laccases are blue-copper phenoloxidases that mainly catalyze the one-electron oxidation of phenolic and non-phenolic components (Gianfreda et al., 1999). We determined laccase activity of the isolated *Tulasnellaceae* spp. to certify whether they can secrete laccases. Our method was based on procedures outlined in Bonugli-Santos et al. (2010) and Fillat et al. (2016), but with some modifications. First, 160 μl 1.0 mM 2,2′-Azinobis-(3-ethylbenzthiazoline-6-sulphonate, ABTS) solution and 20 μl 0.1 mM Sodium tartrate buffer (pH = 5.0) was added to a 96-well plate, shaken and mixed evenly. After adding 20 μl crude enzyme solution into the reaction system, the absorbance at 420 nm was measured immediately, and the increase of absorbance per minute within the initial 3 min was recorded. One unit laccase activity was defined as the amount of enzyme required to oxidize 1 μmol ABTS per milliliter of reaction solution in 1 min.

#### Determination of Superoxide Dismutase

Finally, the activity of the superoxide dismutase (SOD) was determined by measuring the ability of the fungal extracellular enzymes to remove superoxide anions (O_2_^−^). The xanthine and xanthine oxidase system can produce O_2_^−^, which oxidizes nitro tetrazolium chloride blue (NBT) to blue formazan with a maximum absorption peak at 560 nm. SOD competes with NBT to consume O_2_^−^, so that it inhibits formation of blue formazan. The SOD activity is assessed by measuring the inhibition percentage of blue formazan in the reaction system. The method was mainly based on Beauchamp and Fridovich (1971), with some changes. First, 1.16 mM EDTA phosphate buffer solution, 2.24 mM NBT solution, and 1.8 mM xanthine solution in a volume ratio of 138: 5: 17 were mixed to obtain the reaction solution. Next, 160 μl reaction solution and 20 μl 0.05 M potassium phosphate buffer (pH = 7.8) were added to the 96-well plate, followed by stirring and mixing to form the blank system. Then, 0.02 unit/ml xanthine oxidase was added to the blank system and the change of absorbance at 560 nm per minute was measured. In the next step, 160 μl reaction solution and 20 μl crude enzyme solution were added to the 96-well plate, and then stirred and mixed to form the experimental system. The 0.02 unit/ml xanthine oxidase was added into the experimental system to measure the increase of absorbance per minute and the percentage of inhibition was calculated as


$$IP=\frac{\Delta A_{b}-\Delta A_{e}}{\Delta A_{b}}\times 100\%$$

where Δ*A_b_* and Δ*A_e_* represent the increment of absorbance of the blank system and experimental system, respectively. One unit of SOD activity was defined as the amount of SOD present in the solution when the inhibition percentage reaches 50%.

### Determination Preference of Carbon and Nitrogen Sources

To test whether fungi derived from terrestrial or epiphytic environments prefer different resources, the fungal growth rate in media with different carbon and nitrogen sources was determined. The basic culture medium consisted of 20.0 g glucose, 2.0 g yeast extract, 2.0 g peptone, 1.0 g K_2_HPO_4_, 0.46 g KH_2_PO_4_, 0.5 g MgSO_4_·7H_2_O, and 15.0 g agar. Glucose was replaced by other carbon sources using the same amount of carbon, namely 20.0 g galactose, 20.0 g fructose, 20.0 g xylose, 20.0 g arabinose, 20.0 g mannose, 18.2 g rhamnose, 19.0 g cellobiose, 24.2 g carboxymethyl cellulose (CMC), and 18.0 g soluble starch. To assess the preference for different nitrogen sources, the basic medium was used as well, but now peptone was replaced by either 1.32 g ammonium sulfate, 1.32 g (NH_4_)_2_HPO_4_, 1.55 g ammonium acetate, 2.03 g potassium nitrate, 1.38 g sodium nitrite, 0.87 g arginine, 1.47 g lysine, 1.52 g glycine, or 0.60 g urea, respectively. All carbon and nitrogen culture media were sterilized by autoclaving at 122°C for 25 min. After cooling to about 40°C, 100 U/ml penicillin and 100 mg/ml streptomycin were added. According to the volume of 20 ml per dish, the products were poured into a 90 mm sterile petri dish and then cooled and solidified.

The 24 strains of *Tulasnellaceae* spp. were used to compare the carbon/nitrogen preference between terrestrial and epiphytic orchids. Before inoculation, the strains were activated on a PDA medium for 7 days and then transferred to a clear water agar medium for growth for another 7 days. After that, holes were drilled at the edge of fungal colonies with a 5 mm perforator, and the obtained inoculated blocks were inverted in the center of media with the different carbon and nitrogen sources. Each treatment was replicated three times and cultured in darkness at 20°C. When the fungal colony was about to cover four-fifths of the Petri dish, the diameter of the fungal colony was determined by measuring the average maximum colony diameter. Subsequently, colony diameter was divided by the number of days to obtain the average daily growth rate of each fungus-treatment combination.

### Data Analysis

To investigate whether extracellular enzyme activities differed between fungi isolated from terrestrial and epiphytic orchids, a two-way ANOVA was used with the orchid life form, culture time and their interaction included as fixed factors and activities of the different enzymes as dependent variables. In addition, enzyme activities measured at a given day and average daily growth rates were compared between fungi isolated from epiphytic and terrestrial orchids using *t*-tests and Mann-Whitney *U* tests. Unpaired *t*-tests were used when the sample data passed both the normality test (*α* = 5%) and *F* test (*α* = 5%), while Welch’s *t*-test was used when the sample data passed the normality test (*α* = 5%), but not the *F* test (α = 5%). A Mann-Whitney *U* test was used when the sample data did not pass the normality test (*α* = 5%). All analyses were performed using the statistical software GraphPad Prism version 8.0.0 for Windows (GraphPad Software, San Diego, California, United States).

## Results

### Isolation of *Tulasnellaceae* spp.

A total of 135 fungal isolates was recovered from the pelotons of 90 root samples from 18 orchid species that were collected from the subtropical forest in southern China. About 92 isolates, belonging to 24 taxa, were identified as *Tulasnellaceae* spp. (Table 1). The other 43 fungal isolates were identified as other mycorrhizal fungi or fungal endophytes (data not shown). Among the 24 taxa of *Tulasnellaceae* spp., half of them were isolated from terrestrial orchids, while the other half were isolated from epiphytic orchids.

### Extracellular Enzyme Activities

Two-way ANOVA revealed that the activities of all investigated extracellular enzymes were significantly influenced by orchid life form, culture time and their interactions, except for lignin peroxidase, which was not influenced by orchid life form, and xylanase, which was not influenced by the interaction between life form and culture time (Table 2).

**Table 2:** Effect of host plant life form (epiphytic vs. terrestrial) and culture time on extracellular enzyme activity.

Extracellular enzyme	Life form		Culture time		Life form*Culture time	
	*F*	*p*	*F*	*p*	*F*	*p*
Cellulase	18.350	**<0.001**	4.964	**<0.001**	5.723	**<0.001**
Xylanase	19.624	**<0.001**	11.052	**<0.001**	1.680	0.111
Lignin peroxidase	0.883	0.348	3.264	**0.002**	3.651	**0.001**
Laccase	6.481	**0.011**	10.916	**<0.001**	3.074	**0.003**
Superoxide dismutase	11.864	**0.001**	55.292	**<0.001**	5.309	**<0.001**

Bold values are significant at *p* < 0.05.

**Figure 1:**
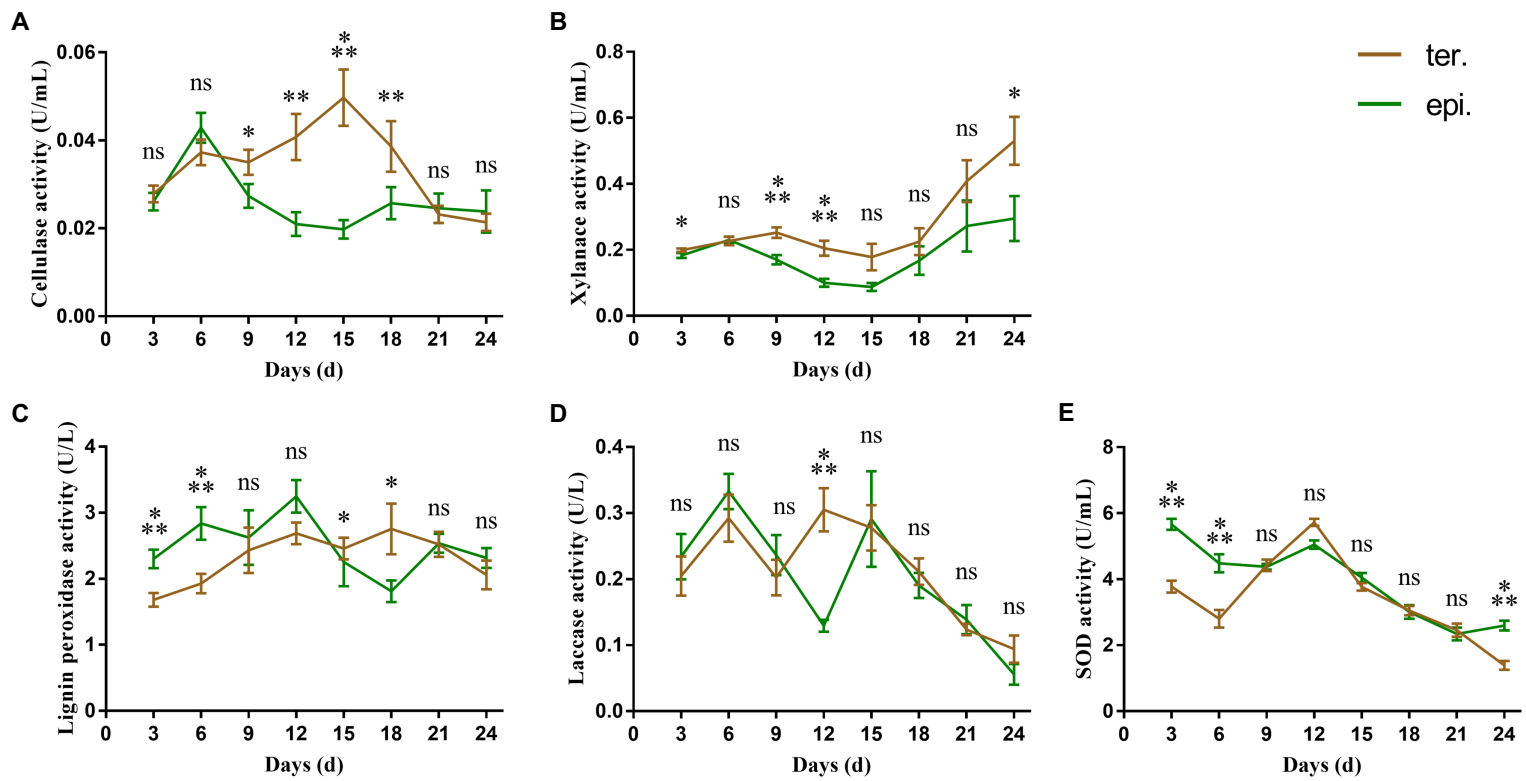
Extracellular enzyme activities of 24 different *Tulasnellaceae* spp. The green line connects the average enzyme activities of epiphytic fungi at different time points, while the brown line connects the average enzyme activities of terrestrial fungi. Error bar represent the SEM. **(A)** cellulase; **(B)** xylanase; **(C)** lignin peroxidase; **(D)** laccase; and **(E)** superoxide dismutase. ns, not significant; ^*^p < 0.05; ^**^p < 0.01; and ^***^p < 0.001.

The highest cellulase activity was 0.050 ± 0.006 U/ml and 0.043 ± 0.003 U/ml in fungi isolated from terrestrial orchids and epiphytic orchids, respectively (Figure 1A). During the 24 days culture period, cellulase activities at day 9, 12, 15, and 18 were significantly influenced by life form (*p* < 0.05; *p* < 0.01; *p* < 0.001; and *p* < 0.01, respectively). Xylanase activity varied between 0.178 ± 0.039 U/ml and 0.531 ± 0.071 U/ml and between 0.088 ± 0.012 U/ml and 0.295 ± 0.067 U/ml in fungi isolated from terrestrial and epiphytic orchids, respectively (Figure 1B). Xylanase activity was significantly higher in fungi isolated from terrestrial orchids than in fungi from epiphytic orchids at day 3, 9, 12, and 24 (*p* < 0.05; *p* < 0.001; *p* < 0.001; and *p* < 0.05, respectively). The highest recorded activity of lignin peroxidase was larger in fungi isolated from epiphytic orchids (3.248 ± 0.242 U/l) than in fungi from terrestrial orchids (2.756 ± 0.376 U/l; Figure 1C). The activity of lignin peroxidase was significantly higher in fungi isolated from epiphytic orchids than in fungi from terrestrial orchids at day 3 and day 6 (all *p* < 0.001).

Overall laccase activity was small, with the average maximum enzyme activity being less than 0.4 U/l. There were two peaks in laccase activity. The first peak emerged on day 6, while the second peak emerged on day 12 and day 15 in fungi from terrestrial and epiphytic orchids, respectively (Figure 1D). Laccase activity differed significantly between fungi from epiphytic (0.130 ± 0.009 U/l) and terrestrial orchids (0.305 ± 0.032 U/l) only at day 12 (*p* < 0.001). SOD activity was significantly higher in fungi from epiphytic orchids than in fungi from terrestrial orchids during the early culture stage (day 3 and day 6, all *p* < 0.001; Figure 1E). The maximum value of SOD activity was 5.663 ± 0.164 U/ml in fungi from epiphytic orchids on day 3, while which was 3.780 ± 0.180 U/ml in fungi from terrestrial orchids.

### Preference of Carbon and Nitrogen Sources

#### Carbon Sources

**Figure 2:**
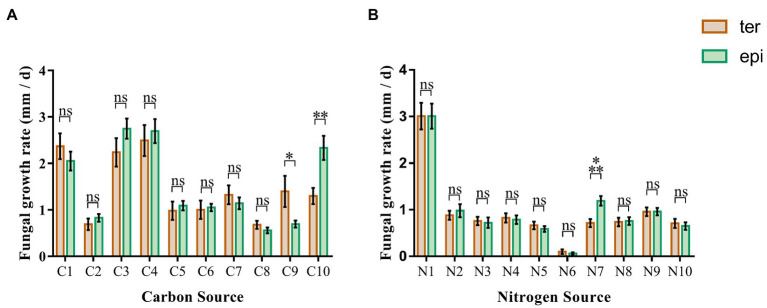
Fungal growth rates in media containing 20 different carbon and nitrogen sources. The green and brown bar represent the average growth rate of epiphytic and terrestrial fungi, respectively. **(A)** carbon sources; **(B)** nitrogen sources. C1, glucose; C2, galactose; C3, fructose; C4, xylose; C5, arabinose; C6, mannose; C7, rhamnose; C8, cellobiose; C9, carboxymethyl cellulose; C10, starch; N1, peptone; N2, ammonium sulfate; N3, diammonium phosphate; N4, ammonium Acetate; N5, potassium nitrate; N6, potassium nitrite; N7, arginine; N8, lysine; N9, glycine; N10, urea; and ns, not significant; ^*^p < 0.05; ^**^p < 0.01; and ^***^p < 0.001.

Carbon utilization of the 24 isolates of *Tulasnellaceae* spp. showed that fungal growth on media containing glucose, fructose and xylose was faster, while growth on media containing cellodisaccharide, galactose, arabinose, and mannose was slower (Figure 2A). There were no significant differences (*p* > 0.05) in growth between fungi isolated from epiphytic and terrestrial orchids. Only when CMC and starch were used as single carbon source, growth performance differed significantly between both types of fungi (*p* = 0.0016, respectively). On a medium with CMC, the growth rate of fungi from terrestrial orchids (1.397 ± 0.337 mm/d) was significantly higher than that of fungi from epiphytic orchids (0.695 ± 0.077 mm/d; *p* < 0.05). When starch was used as the single carbon source, the opposite pattern was observed, the growth rate of fungi from epiphytic orchids (2.331 ± 0.2608 mm/d) being higher than that of fungi from terrestrial orchids (1.300 ± 0.174 mm/d; *p* < 0.01).

#### Nitrogen Sources

The growth rate of *Tulasnellaceae* spp. on peptone, a compound nitrogen source medium, was significantly higher than that on media with a single compound as nitrogen source (Figure 2B). Almost no growth was observed on the medium with potassium nitrite as nitrogen source. Growth rates were similar on media with ammonium sulfate, diammonium hydrogen phosphate, ammonium acetate, potassium nitrate, arginine, lysine, glycine, and urea as nitrogen source. Arginine was the only nitrogen source that led to a significant difference in growth rate between fungi isolated from terrestrial (0.715 ± 0.088 mm/d) and epiphytic orchids (1.190 ± 0.103 mm/d; *p* < 0.001). No significant differences were detected on the other nine nitrogen source media (*p* > 0.05).

## Discussion

Because most OMF are considered saprophytic, it can be assumed that they have the capacity to absorb mineral nutrients from the environment by secretion of extracellular enzymes. Sequencing of the fungal genomes of ericoid, ECM, and orchid mycorrhizal fungi has indeed shown that OMF, in contrast to ectomycorrhizal fungi, have retained an extensive decay apparatus that allows degradation of crystalline cellulose (Kohler et al., 2015). Indirect evidence that OMF can release these extracellular enzymes was provided by Nurfadilah et al. (2013), who showed that OMF were able to grow on liquid media containing cellulose, carboxymethyl cellulose, xylan, and pectin. However, there are still only a few studies that have directly measured extracellular enzymes activities of OMF. For example, cellulase and polyphenol oxidases were used to distinguish isolates of *Ceratorhiza* and *Epulorhiza* (Zelmer et al., 1996; Pereira et al., 2005). In addition, Shubha and Srinivas (2017) reported that endophytic fungi isolated from different organs of *Cymbidium aloifolium* differed in the production of extracellular enzymes.

Because both OMF composition and the availability of nutrient resources are different between epiphytic and terrestrial orchids, it can be assumed that nutrient accessibility and enzyme activities differ between fungi from terrestrial and epiphytic orchids. It has, for example, already been shown that OMF can utilize multiple nutrient sources, such as carbon, nitrogen (organic and inorganic), and phosphorous (organic and inorganic), and that the biomass of OMF in the same culture medium differed between fungal genera (Nurfadilah et al., 2013), suggesting different nutrient-acquisition strategies between different OMF. Mehra et al. (2017) further showed that carbon source utilization by orchid mycorrhizal fungi differed between common and endangered species of *Caladenia* (Orchidaceae) and hence may affect the conservation status of host orchids. Moreover, the patchy distribution and abundance of accessible carbon in natural environments may be one of the reasons why OMF and the orchids that depend on them often show a patchy spatial distribution (Wardle, 1992; McCormick et al., 2012).

Here, we investigated extracellular enzyme activities and nutrient accessibility of fungi isolated from terrestrial and epiphytic orchids. All tested fungi belonged to the family Tulasnellaceae, which has been reported as a major family of fungi associating with orchids (Smith and Read, 2008; Dearnaley et al., 2012). Our results showed that the extracellular enzyme activities differed between fungi isolated from terrestrial and epiphytic orchids. For example, fungi from terrestrial orchids generally displayed significantly higher cellulase activity than fungi from epiphytic orchids, which indicates that fungi from terrestrial species display a stronger ability to degrade cellulose. Cellulose, as the most abundant biological organic substance in nature, is the dominant carbon source for microorganisms (Lynd et al., 2002). Because cellulose is more abundant in terrestrial habitats than in epiphytic habitats, a more efficient degradation of cellulose would be advantageous for fungi growing in terrestrial habitats. Similarly, xylanase, another glycoside-hydrolase to degrade the most abundant component of hemicellulose, showed a higher activity in fungi from terrestrial orchids than in fungi from epiphytic orchids.

In addition to the two extracellular glycoside-hydrolases, we also detected two polyphenol oxidase activities. Although our results suggested higher lignin peroxidase activity in fungi from epiphytic orchids at early culture stages and a higher laccase activity in fungi from terrestrial orchids at the middle culture stage, it is worth noting that both enzyme activities were very low. Kohler et al. (2015) and Miyauchi et al. (2020) sequenced the genome of *Tulasnella calospora* and found that the genes encoding ligninolytic class II peroxidases and laccase were lost in *Tulasnella* genera during their evolution. Based on our enzyme activity data and the existing genomic data of *Tulasnellaceae* spp., it seems that the ability of *Tulasnellaceae* spp. to degrade lignin may be insufficient.

Superoxide dismutase activity was significantly higher in fungi from epiphytic orchids than from terrestrial orchids, suggesting that fungi associating with epiphytic orchids can release higher amounts of this extracellular enzyme than fungi associating with terrestrial orchids. SOD is one of the most important antioxidant enzymes and has also been linked to drought resistance (Asada, 2006). SOD can also release reactive oxygen stress caused by the high light exposure of plants during photosynthesis (Hwang et al., 1999). Epiphytic orchids endure more light exposure and water shortage than terrestrial orchids, although it is still unclear whether OMF play a role in enhancing the ability of stress resistance in the host plant. However, the fungi associating with epiphytic orchids are capable of enduring the light and water conditions that characterize these habitats. Besides, as the primary anti-disease substance of host trees, reactive oxygen is most likely secreted in epiphytic environments (Torres et al., 2006), suggesting that higher SOD activity in fungi associating with epiphytic habitats also protects them from the tree’s chemical attack, which is in line with fungal survival requirements.

In this research, no significant differences were found between fungi from epiphytic and terrestrial orchids in their capacity to utilize monosaccharides. This suggests that both types of fungi have monosaccharide transporters on their cell membranes that can transport various monosaccharide molecules (Helber et al., 2011) and allow these fungi to utilize a more extensive source of nutrients to support their saprophytic lifestyle. However, in terms of polysaccharides (CMC and starch), different patterns were observed. Growth rates of fungi from terrestrial orchids were significantly higher than those of fungi from epiphytic orchids when CMC was added to the medium, whereas the opposite pattern was found for starch. Because cellulase activity was higher in fungi from terrestrial orchids, it can be expected that these fungi can better degrade CMC into monosaccharides and absorb them. Because seeds of terrestrial orchids are less exposed to light than those of epiphytic orchids, terrestrial orchids may associate with specific mycorrhizal fungi that have a stronger ability to absorb carbon sources from the environment, in particular cellulose, which is the main carbon source in natural environment, while epiphytic orchids may associate with fungi that have other functions besides carbon supply. For the higher ability of fungi isolated from epiphytic orchids to utilize starch, we speculate that those fungi have higher amylase activity and can get carbon from the epiphytes’ roots. The ability of orchid mycorrhizal fungi to utilize carbon sources from host orchids has been previously reported (Cameron et al., 2006, 2008). Moreover, it has also been shown that genes related to bidirectional sugar transport are upregulated in the symbiotic protocorms of orchids and OMF (Perotto et al., 2014). The direction of the transport of sugar by this transporter is mainly related to the concentration gradient of sugar (Chen et al., 2010). In the cortex tissue of orchid roots, cells colonized by mycorrhizal fungi did not contain starch grains, but uncolonized cells did (Aybeke et al., 2010). This suggests that fungi can degrade starch grains in cells during colonization. Because of the environmental conditions characterizing the habitats of epiphytic orchids, the photosynthetic capacity of epiphytic orchids is probably higher than that of terrestrial orchids, which may cause epiphytic orchids to have more carbon sources to share with their fungal partners.

Regarding nitrogen sources, Nurfadilah et al. (2013) showed that *Tulasnellaceae* spp. could only use ammonium salts, but not nitrate as inorganic nitrogen sources. However, in our study, *Tulasnellaceae* spp. could also grow on a potassium nitrate medium in addition to ammonium salts. However, no growth was observed when nitrite was used as a nitrogen source, perhaps because the genomes of the Tulasnellaceae fungi do not contain genes encoding for nitrite transporters (2.A.1.8.5).^1^ Arginine was the only nitrogen source showing a significant difference in growth between fungi from epiphytic and terrestrial orchids. In arbuscular mycorrhiza (AM), arginine is the most important amino acid in the extra-root hyphae, accounting for 70–90% of the total free amino acids, and the main carrier for nitrogen storage and transportation material (Johansen et al., 1996; Govindarajulu et al., 2005). In intracellular hyphae, arginase, and urease, which are involved in the hydrolysis of arginine and the urea cycle, are highly expressed to hydrolyze arginine into NH_4_^+^, which is then transported to host plants (Govindarajulu et al., 2005). In ECM, arginine is also the primary nitrogen material of fungi, and then hydrolyzed into urea and NH_4_^+^ by arginase and urease at the symbiotic interface (Russnak et al., 2001). In orchid mycorrhiza, Fochi et al. (2017) reported that argininosuccinate lyase was significantly upregulated during the symbiosis between *Tulasnella calospora* and *Serapias vomeracea* and that this enzyme plays an important role in arginine synthesis. However, unlike AM and ECM, arginase and urease were not upregulated or even downregulated in orchid mycorrhizal symbiosis, suggesting that the transfer of nitrogen from fungi to orchids may be in the form of organic nitrogen, such as amino acids (Dearnaley and Cameron, 2016; Fochi et al., 2017). In this study, fungi from epiphytic orchids had a stronger ability to assimilate arginine than fungi from terrestrial orchids, which may increase arginine reserves in fungi of epiphytic orchids and potentially a higher nitrogen supply.

Previous research has shown that the expression of extracellular enzymes can be influenced by culture conditions and substrates (Arora and Gill, 2001; Cullings et al., 2008; Hadibarata and Yuniarto, 2020), and therefore, the differences in extracellular enzyme activities that were observed here between fungal groups or between fungi isolated from different life forms need to be interpreted with caution and most likely cannot be directly extrapolated to other systems. Moreover, under natural conditions, many orchids interact with more than one OMF, and some OMF are unculturable and only detected by molecular analyses. Therefore, biochemical assays of extracellular enzyme activities and carbon/nitrogen resources preference were only performed for fungi that could be cultured. To get better insights into extracellular enzyme activities and carbon/nitrogen preference of non-culturable fungi, DNA sequences for extracellular enzymes and carbon/nitrogen resources transporters from other fungi could be used to design primers to quantify the mRNA transcripts in orchid mycorrhizal associations, and to draw more precise conclusions.

## Conclusion

This study investigated extracellular enzyme activities and carbon/nitrogen preference in OMF associated with different life forms of orchids. Our results indicated that extracellular enzyme activities and to a lesser extent carbon/nitrogen preference differed between OMF isolated from terrestrial and epiphytic orchids, which may indicate functional differentiation and ecological adaptation of OMF to local growth conditions.

## Data Availability Statement

The original contributions presented in the study are included in the article/supplementary material, further inquiries can be directed to the corresponding author.

## Author Contributions

XX and HJ conceived and designed the study. ZZ, SS, and NL performed the experiments. XX and QL collected the orchid root samples. ZZ performed the statistical analyses and wrote the first draft of the manuscript. All authors contributed to the article and approved the submitted version.

## Funding

This research was financially supported by CAMS Initiative for Innovative Medicine (no. 2021-1-I2M-031) and the National Natural Sciences Foundation of China (32170013).

## Conflict of Interest

The authors declare that the research was conducted in the absence of any commercial or financial relationships that could be construed as a potential conflict of interest.

## Publisher’s Note

All claims expressed in this article are solely those of the authors and do not necessarily represent those of their affiliated organizations, or those of the publisher, the editors and the reviewers. Any product that may be evaluated in this article, or claim that may be made by its manufacturer, is not guaranteed or endorsed by the publisher.

## Footnotes

^1^ https://mycocosm.jgi.doe.gov/Cantharellales/Cantharellales.info.html
